# Demography and Life History of the Egg Parasitoid, *Trichogramma brassicae*, on Two Moths *Anagasta kuehniella* and *Plodia interpunctella* in the Laboratory

**DOI:** 10.1673/031.009.5101

**Published:** 2009-07-10

**Authors:** S Iranipour, A Farazmand, M Saber, Jafarloo M Mashhadi

**Affiliations:** ^1^ Department of Plant Protection, Faculty of Agriculture, University of Tabriz, Tabriz, Iran; ^2^ Department of Plant Protection, Faculty of Agriculture, University of Maragheh, Maragheh, Iran; ^3^ Agriculture and Natural Resource Research Center of East Azarbaidjan, Tabriz, Iran

**Keywords:** intrinsic rate of natural increase, net replacement rate, fertility-life table

## Abstract

The egg parasitoid, *Trichogramma brassicae* Bezdenko (Hymenoptera: Trichogrammatidae) is the most important and widely distributed species of *Trichogramma* in Iran. It attacks eggs of several lepidopterous pests, and is a major biological control agent. Rearing parasitoids is necessary for experimental work, and, potentially, for mass release in the field. Selecting a suitable host is critical for developing a successful rearing method. If other conditions are the same, the rate of population increase will be a suitable indicator of parasitoid performance on different hosts. However, conclusions based on a single generation can be misleading because of the learning ability of parasitoids. Life history parameters of *T. brassicae* were studied on two hosts easily reared in the laboratory, *Anagasta kuehniella* Zeller, and *Plodia interpunctella* Hübner (Lepidoptera: Pyralidae). All the experiments were carried out at 24 ± 1°C, 65±10% RH, and 16:8 L:D photoperiod. Eight parameters including gross and net reproductive rates (GRR and R_0_ respectively), intrinsic rate of natural increase (r_m_), finite rate of population increase (λ), intrinsic birth and death rates (b and d respectively), cohort generation time (T), and doubling time (DT) were compared between two hosts for two generations. All parameters showed a highly significant difference (α = 0.01) between hosts. GRR, R_0_, r_m_, λ, and b were higher, while d, T, and DT were lower in *Anagasta* than *Plodia*. The intrinsic rate of natural increase was 0.2912 and 0.2145 female/female/day and net replacement rate was 45.51 and 19.26 female/female/generation in *Anagasta* and *Plodia* respectively. Differences between generations were significant except for r_m_, λ, and d. The net replacement rate was 28.56 and 39 in the 1^st^ and 2^nd^ generations respectively. These results showed that *A. kuehniella* was a better host than *P. interpunctella*. Higher reproduction occurred in the second generation that may be due to increased adaptation to experimental conditions.

## Introduction

Insect eggs belonging to 11 orders are attacked by different species of Trichogrammatidae parasitoids. Lepidopterous insects are the most preferred hosts (Sorokina 1999). Augmentation of parasitoids is used against some serious pests throughout the world (Li 1994; Smith 1996). *Trichogramma brassicae* Bezdenko (Hymenoptera: Trichogrammatidae) is the most important and widely distributed species of *Trichogramma* in Iran (Ebrahimi et al. 1998). Many *Trichogramma* species can be easily reared on laboratory hosts such as *Anagasta kuehniella, Sitotroga cerealella, Plodia interpunctella, Galleria mellonella*, and *Corcyra cephalonica* (Smith 1996; Shojai et al. 1998; Ebrahimi 2004). Furthermore, major crop pests such as the rice stem borer *Chilo suppressalis*, the European corn borer, *Ostrinia nubilalis*, the cotton bollworm *Helicoverpa armigera*, and some related species are target hosts of *T. brassicae* and related species (Li 1994; Dutton et al. 1996; Greenberg et al. 1998; Ebrahimi et al. 1998).

Success in biological control by *Trichogramma* depends on identification of the best host species, as well as a good understanding of the ecological requirements of the parasitoid wasp (Parra et al. 1987; van Lenteren et al. 1997). One of the most important aspects in ecology of the parasitoid is a suitable host. The size and age of the species chosen are important characteristics that determine the quality of a host (Hiehata et al. 1976; Houseweart et al. 1982; Bournier 1982; Brower 1983; Ahmad and Sivapragasam 1984; Pak 1986; Lewis and Redlinger 1989; Hintz and Andow 1990; Reznik and Umarova 1990; Ruberson and Timothy 1992; Monje et al. 1999; Jeffry and Robert 2000; Mansfield and Mills 2002; Roriz et al. 2006; El-Wakeil 2007). There are a few ecological studies on local populations in Iran that show trichogrammatids are affected by these factors (for example Karimian 1999; Attaran et al. 2000; Dadpour Moghanlou 2002; Haghani and Fathipour 2004; Alizadeh and Ebrahimi 2004; Karimi Malati et al. 2004; Shirazi 2004; Hosseini Bai et al. 2006). However demographic traits such as the intrinsic rate of natural increase or net reproductive rate have been estimated only in a few studies (Dadpour Moghanlou 2002; Haghani and Fathipour 2004). According to Andrewartha and Birch (1954) demographic parameters are the best indicators of fitness of a population and are suitable criteria for comparing physiological states of different species, populations, etc. or even as bioclimatic or nutritional indices (Messenger 1964; Dent and Walton 1997). Also, in biological control programs, population growth rate is an essential criterion for preliminary screening and choice of potential biocontrol agents (van Lenteren and Woets 1988).

To achieve a successful laboratory culture of *T. brassicae* it is necessary to study the effects of different ecological factors on important biological parameters of local populations of *T. brassicae*. As van Driesche and Bellows (1996) point out, rearing natural enemies on natural hosts is often difficult and expensive. Augmentation using alternative hosts is therefore usually necessary. Stored products moths often have been used traditionally for rearing *Trichogramma* species (Smith 1996). *S. cerealella, A. kuehniella*, and *P. interpunctella* are not only alternative hosts but also obvious target pests (Smith 1996; Shojai et al., 1998; Ebrahimi 2004). Availability due to worldwide distribution, as well as an inexpensive rearing method (Sepasgozarian 1966; Behdad 2002) are the most important advantages of these species for mass production purposes.

Natural enemies are able to learn (Bigler 1994; van Driesche and Bellows 1996). This ability allows them to improve their response to their host when they are reared sequential generations on a host (van Driesche and Bellows 1996). This occurs via enhancing their skills in orientation, host finding, host detection and acceptance (Noldus et al. 1990). Van Bergeijk et al. (1989) observed different responses in *T. brassicae* to European corn borer when it was reared previously on the same host compared to *A. kuehniella*.

In this study, life history statistics are examined for *T. brassicae* on two laboratory hosts, *A. kuehniella*, and *P. interpunctella* in order to explore if there is any advantage to each one. As the null hypothesis we assumed that no differences were present between these hosts so that both are similar in quality for their host. If this is the case, then either host can be used equally well for production purposes, assuming all other conditions are the same. Rejection of the null hypothesis means that the hosts are not of the same value for rearing and the best host can be used. As mentioned earlier, continuous rearing on a host can improve the impact of a parasitoid. If this is the case, then considering only one generation in host evaluation may be misleading. For example if continuous rearing on a host leads to improvement in parasitoid performance on one of the hosts, then it may be recommendable using it in sequential cultures in spite of its initial low performance. Thus the second null hypothesis is that the parasitoid has equal performance in sequential generations.

## Materials and Methods

### Host cultures

A culture of the Indian meal moth, *Plodia interpunctella* Hübner (Lepidoptera: Pyralidae), was reared on pistachio in the Department of Plant Protection, Faculty of Agriculture, University of Tabriz. To adapt the culture to the laboratory conditons it was reared two generations on single cross hybrid of maize kernels at 24±1°C, 65±10% RH, and 16:8 L:D photoperiod in an incubator. Plastic rectangular containers (201×4×6 cm) were used for rearing larvae.

A culture of the flour moth, *Anagasta kuehniella* Zeller(Lepidoptera: Pyralidae) was reared on wheat flour in the same location. It was reared two generations on wheat flour cultivar Omid under the conditions described above. Similar containers were used for this purpose.

Cylindrical containers (30cm in height, 11 cm in diameter), were used to confine moths to obtain eggs. Ten pairs of adult moths were removed by an aspirator and transferred to a cylindrical dish. The top of the dish was covered by a 10 mesh cloth net and set upside down on a piece of paper. Deposited eggs were collected every day and used in experiments.

### Preparation of parasitoids for experiments

The *Trichogramma brassicae* used in this study, were collected from eggs of the cereal moth, *Sitotroga cerealella* (Olivier) (Lepidoptera: Gelechiidae), obtained from the Agricultural Research Center of East Azerbaijan, Tabriz. These wasps had been already reared on the *S. cerealella* for five generations. To adapt it to experimental conditions it was reared for two generations under the conditions described above using *S. cerealella* eggs as the host. Honey droplets were used to feed wasps.

One-day-old eggs of both hosts were offered to newly emerged females of *T. brassicae* for 24 hours. Female wasps were then removed from host eggs and they were held under the same conditions until symptoms of parasitism appeared. Forty parasitized eggs from each host were selected randomly and the parasitoids were reared to adults. Each surviving female was then confined with a male that had been reared under the same conditions. Males were replaced when they died. Fifty eggs of the same host species that parents had been reared on were offered daily to each pair of wasps up to the time of death of the female. The number of eggs offered daily to the wasps was determined based on a functional response experiment under the same conditions (Farazmand and Iranipour 2006) in which the maximum attack rate was 40 hosts/wasp/day obtained for *A. kuehniella*. Cohorts of the second generation were selected at the peak of oviposition.

**Table 1.**
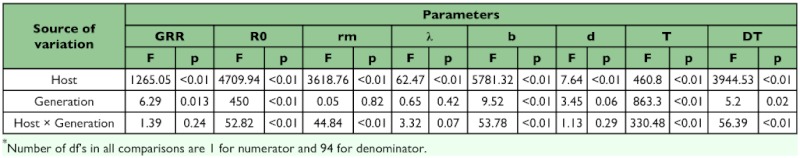
Summary of analysis of variance (F and p-values) for eight life history parameters of *T. brassicae* on two host species *A. kuehniella*, and *P. interpunctella* for two generations^*^

### Experimental design and data analyses

The experiment was designed as a 2×2 factorial with completely randomized design. One of the factors was host with two levels *A. kuehniella* and *P. interpunctella*. The other factor was generation with two levels. Due to unequal mortality among treatments, the analysis was converted to an imbalanced design. Eight parameters including gross reproductive rate (GRR), net replacement rate (R_0_), intrinsic rate of natural increase (r_m_), finite rate of increase (λ), intrinsic birth rate (b), intrinsic death rate (d), cohort generation time (T), and doubling time (DT) were calculated as described by Carey (1993). Variances and standard errors were measured using the jackknife method of Meyer et al. (1986). Analyses of variances and comparisons of means were carried out using SAS software (SAS Institute Inc.). Survivorship curves were drawn using data in column lx and type was determined by entropy (Demitrius 1978; Carey 1993).

## Results

A summary of the analyses of variances for the eight above-mentioned statistics are shown in table 1. All the parameters differ highly significantly (p <0.01) between hosts, as GRR, R_0_, r_m_, λ, and b are higher while d, T, and DT are lower in *A. kuehniella* than *P. interpunctella* (Table 2). These results reveal that *A. kuehniella* is a more desirable host than *P. interpunctella* for *T. brassicae*. There are also significant differences in all parameters except for rm, λ, and d in the second generation. All parameters but doubling time were higher in second generation (Table 2). Because R_0_ and T have opposite effects on r_m_, the latter parameter showed no significant difference in the two generations. These data suggest that *T. brassicae* has habituated to hosts as well as other experimental conditions during the first generation. Interactions between two factors were significant in all but three parameters (GRR, λ and d). This means that level of adaptation during the second generation was not equal between the two hosts. Components of life history statistics in each host and generation are shown in Table 3.

**Table 2.**
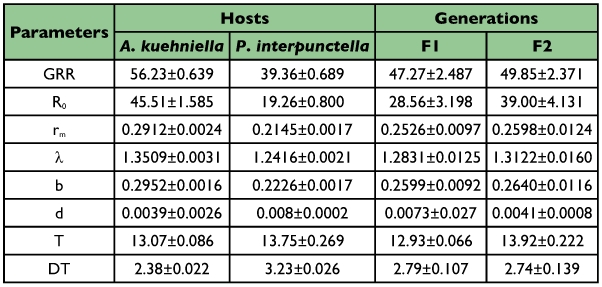
Summary statistics of life history of *T. brassicae* on two hosts in two generations (mean ± 95% confidence interval).

**Table 3.**
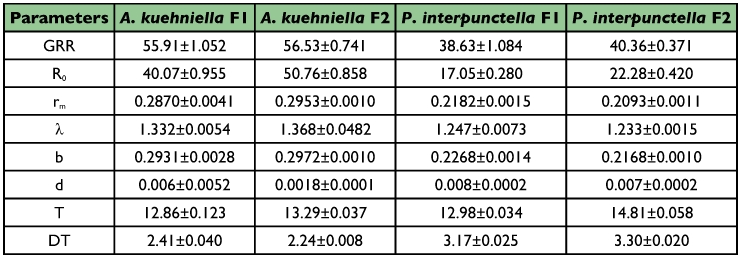
Life history statistics of *T. brassicae* in each host and generation separately (mean ± 95% confidence interval).

### Gross reproductive rate

The gross reproductive rate (GRR) was higher in *A. kuehniella* than *P. interpunctella* by a factor of 1.4 (F = 1265.1, P <0.01) (Table 2).

### Net reproductive rate

The net reproductive rate (R_0_) of *A. kuehniella* was more than twice as high as *P. interpunctella* (F = 4709.9, P <0.01). A 36.55% increase occurred between the first and second generations (Table 2).

### Intrinsic rate of natural increase

The intrinsic rate of natural increase (rm) was significantly higher for *A. kuehniella* than *P. interpunctella* (F = 3618.8, P<0.01). There was no significant difference between generations (F = 0.05, P = 0.82) (Table 2).

### Finite rate of population increase

The finite rate of population increase (λ) is calculated easily from the intrinsic rate of natural increase. Therefore its changes follow the same pattern.

### Intrinsic birth rate and death rate

The balance between these statistics (b and d) determines population growth rate. They changed in an inverse direction, as the birth rate was higher (F = 5781.3, P <0.01) and the death rate was less (F = 7.64, P <0.01) in *A. kuehniella* than *P. interpunctella*. So the intrinsic rate of natural increase (r_m_) was higher in *A. kuehniella* (Table 2). The intrinsic birth rate also was higher in second generation (F = 9.52, P <0.01), while the intrinsic death rate was similar in both (F = 3.45, P = 0.06) (Tables 2 and 3).

### Cohort generation time

Cohort generation time (T) was significantly longer in *P. interpunctella* than *A. kuehniella* (F = 460.8, P <0.01). There was also a significant difference between the two generations (F = 863.3, P <0.01), as in second one was one day more (12.93 ± 0.066 vs. 13.92 ± 0.222) (Table 2). However, the differences were small and significance of such small differences means a low variance in this statistic due to the limited reproductive period.

### Doubling time

In *T.brassicae* the population doubles every 2.38 ± 0.022 days for *A. kuehniella* and every 3.23±0.026 days for *P. interpunctella*. The differences were significant (F = 3944.5, P <0.01). The difference in doubling time between generations was small but significant (F = 5.2, P = 0.02) (Table 2).

**Figure 1.**
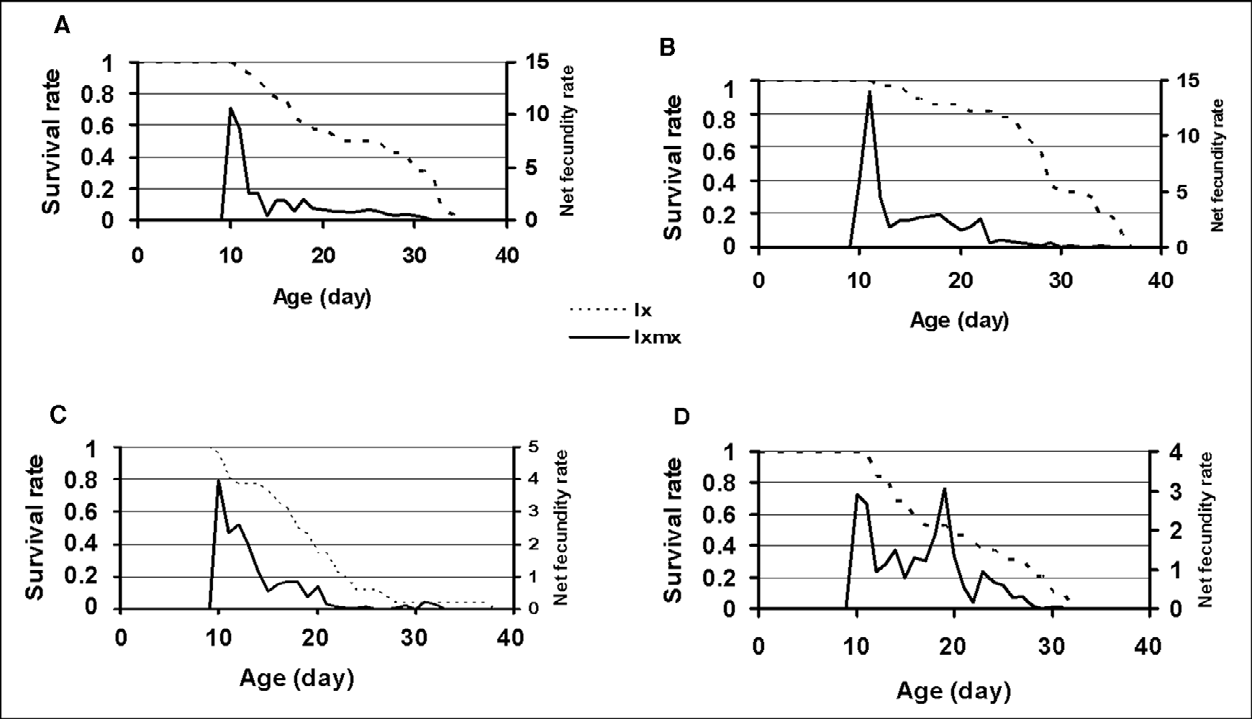
Survivorship curves and net fecundity rates in *Trichogramma brassicae*. A) F1 on *A. kuehniella*, B) F2 on *A. kuehniella*, C) F1 on *P. interpunctella,* D) F2 on *P. interpunctella*

### Survivorship curves

A survivorship curve similar type I of Slobodkin (1980) was observed in all treatments (Figure 1). In order to determine curve type, entropy (Carey 1993) was used as a criterion. An entropy amount below 0.5 shows a survivorship curve near to type 1. During two generations, entropy was 0.28 and 019 in *A. kuehniella*, and 0.31 and 0.30 in *P. interpunctella*. Both survivorship and age specific fecundity are shown in Figure 1.

## Discussion

The results of this study revealed that *A. kuehniella* is a more acceptable host for *T. brassicae* than *P. interpunctella*. A higher GRR, R_0_, r_m_, λ, and b suggest higher reproduction of *T. brassicae* on *A. kuehniella*, whereas lower d, T, and DT may be due to an accelerated development of *A. kuehniella*. Differences in total fecundity, developmental time and other statistics related to life history have observed among hosts in different *Trichogramma* species (e.g. Shirazi 2004; Hosseini Bai 2006; Roriz et al. 2006; El-Wakeil 2007). Shirazi (2004) found a shorter developmental time, and a higher daily fecundity in females of *Trichogramma chilonis* on *Corcyra cephalonica* compared to its natural host, *Helicoverpa armigera*. Larger *T. brassicae* females with higher fecundity were obtained when reared on the cereal moth compared to the Indian meal moth (Hosseini Bai et al. 2006). An average of 34.55 and 21.61 eggs were laid under non-feeding conditions respectively. Significant differences in fecundity and developmental time were observed in *Trichogramma cordubensis* on different noctuid hosts (Roriz et al. 2006). El-Wakeil (2007) also found a higher longevity, parasitism and emergence rates on target host *H. armigera* than three factitious hosts.

The gross and net reproductive rates, and intrinsic rate of natural increase in *T. brassicae* that were found in the present study are well in the range of *Trichogramma embryophagum* and *Trichogramma pintoi* respectively as reported in the Haghani and Fathipour (2004) and Dadpour Moghanlou (2002) studies. GRR, R_0_ and r_m_ in *T. embryophagum* were 55.24, 48.88, and 0.238 reared on the flour moth, *Ephestia kuehniella* and 41.74, 37.63, and 0.218 when reared on *S. cerealella* respectively (Haghani and Fathipour 2004). On the other hand, the above mentioned parameters in *T. pintoi* were estimated to be 46.30, 45.30, and 0.257 when reared on *A. kuehniella*, and 49.19, 45.68, and 0.281 when reared on *S. cerealella* respectively (Dadpour Moghanlou 2002).

Pratissoli and Parra (2000) calculated these statistics for *Trichogramma pretiosum* and *Trichogramma acacioi* in five constant temperatures, 15, 20, 25, 30 and 35°C. In those temperatures, R_0_ was determined to be 13.98, 39.44, 31.53, 54.97, and 15.54 for *T. pretiosum* and 11.85, 62.89 20.64 20.42, and 9.36 for *T. acacioi* at each temperature respectively. Furthermore, r_m_ was calculated as 0.05, 0.21, 0.32, 0.47, and 0.36 in former species while it was 0.05, 0.22, 0.35, 0.34, and 0.30 in later species at the same temperatures respectively. Their results do not resemble *T. brassicae* in this study totally, but there is some similarity at intermediate temperatures (20–30°C). For example R_0_ in *T. acacioi* at 25 and 30 °C, as well as rm in both species at 20 °C is near to their values in *T. brassicae* on *P. interpunctella* in the present study. Haile and Hassan (1999) found an intrinsic rate of increase of 0.309 in *Trichogramma bournier* reared on *S. cerealella* that is very near to *T. brassicae* reared on *A. kuehniella* in our study.

Birth rates in *T. embryophagum* and *T. pintoi* found by Haghani and Fathipour (2004) and Dadpour Moghanlou (2002) were similar to *T. brassicae* in this study, while death rates were a little higher in our study. Both T and DT in our study resemble *T. pintoi* found by Dadpour Moghanlou (2002), as one generation took 14.81 and 13.59 days on *E. kuehniella* and *S. cerealella* respectively (Dadpour Moghanlou 2002). It was considerably longer (16.37–16.49 days) in *T. embryophagum* (Haghani and Fathipour 2004). Doubling time in their studies was between 2.46 and 3.77 days in different experiments. Differences observed in all the statistics between the present study and those of the other studies may be due to variation in species and population sources of parasitoid and/or hosts, rearing background, nutritional state, physical conditions and even analytical approaches.

Lower entropy can also result in a higher survival rate when *T. brassicae* is reared on *A. kuehniella*. On the other hand, a significant increase in GRR, R_0_, and b may due to increased reproduction and adaptation of *T. brassicae* to hosts following one generation of rearing. This is in full agreement with van Bergeijk et al. (1989). It should not to be forgotten that the degree of adaptation is not equal in the two hosts and this is why interaction between host and generation was significant in reproductive rates. Absolute change in R_0_ was higher in *A. kuehniella*. Indeed, the net replacement rate increased significantly with generation whereas r_m_ did not suffer parallel changes. This suggests a trade off between R_0_ and T. In other words generation time lengthened while fecundity increased and population growth rate remained unchanged.
